# Interaction between alcohol drinking and obesity in relation to colorectal cancer risk: a case-control study in Newfoundland and Labrador, Canada

**DOI:** 10.1186/1471-2458-12-94

**Published:** 2012-02-01

**Authors:** Jinhui Zhao, Yun Zhu, Peizhong Peter Wang, Roy West, Sharon Buehler, Zhuoyu Sun, Josh Squires, Barbara Roebothan, John R McLaughlin, Peter T Campbell, Patrick S Parfrey

**Affiliations:** 1 Division of Community Health and Humanities, Faculty of Medicine, Memorial University of Newfoundland, 300 Prince Philip Drive, St. John's, NL, A1B 3V6, Canada; 2 School of Public Health, Tianjin Medical University, 22 Qi Xiangtai Road, Tianjin, 300070, China; 3 Samuel Lunenfeld Research Institute, Mount Sinai Hospital, 60 Murray Street #L5-211, Toronto, ON, M5T 3L9, Canada; 4 Epidemiology Research Program, American Cancer Society, 250 Williams Street NW, Atlanta, GA, 30303, USA; 5 Clinical Epidemiology Unit, Faculty of Medicine, Memorial University of Newfoundland, 300 Prince Philip Drive, St. John's, NL, A1B 3V6, Canada

**Keywords:** Case-control study, Alcohol, Obesity, Colorectal cancer, Interaction, Lifestyles, Newfoundland

## Abstract

**Background:**

While substantive epidemiological literature suggests that alcohol drinking and obesity are potential risk factors of colorectal cancer (CRC), the possible interaction between the two has not been adequately explored. We used a case-control study to examine if alcohol drinking is associated with an increased risk of CRC and if such risk differs in people with and without obesity.

**Methods:**

Newly diagnosed CRC cases were identified between 1999 and 2003 in Newfoundland and Labrador (NL). Cases were frequency-matched by age and sex with controls selected using random digit dialing. Cases (702) and controls (717) completed self-administered questionnaires assessing health and lifestyle variables. Estimates of alcohol intake included types of beverage, years of drinking, and average number of alcohol drinks per day. Odds ratios were estimated to investigate the associations of alcohol independently and when stratified by obesity status on the risk of CRC.

**Results:**

Among obese participants (BMI ≥ 30), alcohol was associated with higher risk of CRC (OR: 2.2; 95% CI: 1.2-4.0) relative to the non-alcohol category. Among obese individuals, 3 or more different types of drinks were associated with a 3.4-fold higher risk of CRC relative to non-drinkers. The risk of CRC also increased with drinking years and drinks daily among obese participants. However, no increased risk was observed in people without obesity.

**Conclusion:**

The effect of alcohol of drinking on CRC seems to be modified by obesity.

## Background

Colorectal cancer (CRC) is one of the most common gastrointestinal tract neoplasms in the world accounting for approximately 9% of all new cancer cases [1]. Incidence rates of CRC vary geographically, being most common in Western countries and less common in Asia and Africa [1]. In Canada, there is substantial variation in CRC incidence and mortality rates among provinces [2]. Very high incidence and mortality rates have been observed in the Atlantic provinces, particularly in Newfoundland and Labrador (NL), where CRC incidence and mortality rates are approximately twice as high as they are in British Columbia and Alberta [2]. It is unclear why such variation exists, but it has been speculated that lifestyle may play an important role in CRC carcinogenesis [2].

Alcohol is one of the best known but most avoidable lifestyle behaviors related to CRC [3,4]. Many epidemiological studies [5-10], but not all [11], have reported a positive association between alcohol consumption and CRC occurrence. A recent meta-analysis of 27 cohort and 34 case-control studies observed a modestly elevated risk of CRC comparing the moderate (12.6-49.9 g/day; RR:1.21) and heavy drinkers (≧ 50 g/day; RR:1.52) with non-/occasional drinkers [3]; the effects also showed a clear dose-response relationship. Another pooled analysis of 8 cohort studies across North America and Europe supported this association by reporting a risk that is as much as 1.41-fold higher among regular high alcohol drinkers (≧ 45 g/day), compared with nondrinkers [12]. However, despite the overall positive association between alcohol drinking and CRC, considerable variations have existed among individual studies, which may not be fully explained by random errors. In recent years, obesity has been found to be correlated with a lower level of glutathione S-transferase A4 (GSTA4) [13], which is an important enzyme responsible for the depredation of acetaldehyde (an established carcinogen metabolized from alcohol) [14]. Hence, we would expect to find an effect modification of alcohol by obesity status and hypothesize that the association between alcohol drinking and CRC may vary across populations.

Historically, the province of NL has a higher alcohol use and obesity prevalence compared with other regions (~1/3 of adults are obese, defined by their body mass index, BMI) [15-17]. However, as yet there has been no study conducted in NL population to investigate the relationship between alcohol consumption and CRC risk, and very little has been reported on the joint effects of alcohol consumption and obesity on CRC risk. The objective of this study is to examine if alcohol drinking is associated with an increased risk of CRC and if such risk differs in people with and without obesity in NL population.

## Methods

### Study participants

CRC patients newly diagnosed in 1999-2003, aged 20-74 years were eligible for inclusion using the Newfoundland Cancer Registry (NCR). NCR records were reviewed to identify cases, and pathology reports were then used to verify the diagnosis according to the International Classification of Diseases 9^th ^revision and 10^th ^revision. Eligible cases were contacted through their attending or family physician to inform them of this study; only those who indicated voluntary participation were recruited. Controls were frequency matched with cases by sex and age (5-year strata), aged 20-74 years, selected from the NL population using random digit dialing (RDD). Briefly, random telephone numbers were generated by SPSS software based on a telephone roll provided by Aliant (a local telephone company in NL). Initial contacts were made through dialing those numbers in a sequential order until the desired number of controls was obtained. A detailed description of the recruitment of controls is reported elsewhere [18-20]. In total, 1,171 cases (84.4% of eligible cases) consented to participate in the study and were sent study materials. Of those, 702 participants (59.9%) completed and returned the questionnaires. A total of 1,602 eligible controls (78.9% of eligible controls) identified using RDD consented verbally to participate in the survey. However, only 717 controls returned the survey packages and the signed consent forms at the end of December 2006, resulting in a participation rate of 44.8%.

### Exposure data

All participants completed three questionnaires: a Personal History Questionnaire (PHQ), a Family History Questionnaire (FHQ), and a Food Frequency Questionnaire (FFQ) [3,4]. The FFQ utilized is a similar but modified version of the validated Hawaii FFQ to include endemic foods in Newfoundland (e.g., salted/pickled meat and smoked/pickled fish) [19,20]. All questionnaires were mailed to participants with self-addressed stamped envelopes once the consent for participation was obtained. If a participant was unable to return finished questionnaires within 3 weeks, a follow-up telephone call was made to ensure that the study package had been received. A telephone interview or assistance was offered when needed. Information was collected on participants' alcohol use in each decade of life since age 20 years. Participants were asked about duration and frequency of 12 oz cans or bottles of beer, 4 oz glasses of wine, 1 oz serving of fortified wine, or 1 oz shots of liquor or spirits consumed per day or per week (see Table 1).

**Table 1:** Questions used to investigate alcohol consumption in respondent's 20 s, and the same questions were asked in respondent's 30 s and 40 s, and 50 s

Q1.	In your 20s, did you ever consume beer or hard cider (at least 3% alcohol) at least once a week for 6 months or longer? • yes • no • don't know
Q1a.	For how many years? ___ years consumed

Q1b.	During those years, how much did you typically consume? ___ number of 12 ounce cans or bottles • per day • per week • don't know

Q2.	In your 20s, did you ever consume wine at least once a week for 6 months or longer? • yes • no • don't know

Q2a.	For how many years? ___ years consumed

Q2b.	During those years, how much did you typically consume? ___ number of 4 ounce glasses of wine • per day • per week • don't know

Q3.	In your 20s, did you ever consume sake, sherry, or port at least once a week for 6 months or longer? • yes • no • don't know

Q3a.	For how many years? ___ years consumed

Q3b.	During those years, how much did you typically consume? ___ number of 1 ounce servings • per day • per week • don't know

Q4.	In your 20s, did you ever consume spirits, liquor mixed drinks, brandy, or liqueurs at least once a week for 6 months or longer? • yes • no • don't know

Q4a.	For how many years? ___ years consumed

Q4b.	During those years, how much did you typically consume? ___ number of 1 ounce shots of liquor or spirits • per day • per week • don't know

Q5.	When you were in your 20s, how many years in total did you consume at least one alcoholic beverage (of any type) a week? ___ years consumed • never consumed alcohol

Q6.	On average, how many alcoholic beverages a week did you consume during those years? That is, how many 4 ounce glasses of wine or 12 ounce cans or bottles of beer or hard cider, or 1 ounce servings of sake, sherry, port, or spirits, mixed drinks and cocktails. ___ number of alcoholic beverages a week • never consumed alcohol

Participants were classified as alcohol drinkers if they ever consumed any alcoholic beverages (e.g. beer, wine and spirits) once a week for 6 months or longer. Others were classified as non-drinkers. Derived variables on alcohol consumption in the analysis included types of alcoholic beverage (0, 1-2, 3+), number of drinking years (0, 1-19, 20+) and average number of drinks daily (0, 1-2(13.5-27.0 g), 3-4(40.5-54.0 g), 5 + (>67.5 g)). Respondents were classified into non-obese (BMI < 30) and obese (BMI ≥ 30) categories [4]. BMI was estimated based on the height and weight questions, "About how tall are you, without your shoes on?" and "How much did you weigh about 1 year before your recent cancer diagnosis (cases) or this survey (controls)?"

Covariates included in the model were age, sex, marital status, education attainment, rural/urban area, and census division. Urban and rural residences were determined based on the definition of rural postal codes [21]. Other variables considered in the analyses were family CRC history (yes/no), other personal cancer history (yes/no), physician diagnosed diabetes (yes/no), physician diagnosed hypercholesterolemia (yes/no), cigarette smoking (ever smoke, never smoke), regular use of aspirin (yes/no), intake of fruits, vegetables and red meats, leisure time physical activity, lifetime use of bulk-forming laxatives and other laxatives (yes/no) and lifetime use of calcium pills or tablets and calcium-based antacids (yes/no). The selection of confounding factors was based on literature/previous studies and biological plausibility.

### Statistical analyses

A descriptive analysis was conducted to show the distribution of sociodemographic characteristics in study populations stratified by case and control status [17]. Comparisons between categorical groups were analyzed using Pearson's chi-square test. The independent association between alcohol intake and risk of CRC was estimated using the odds ratio (OR) and 95% confidence intervals (CI) as an estimate of the relative risk from multivariate logistic regression models, adjusted for potential clustering and confounding [22-24]. Based on univariate logit analysis of the pooled data set, any variable whose univariate test had a *p*-value < 0.20 was considered as a candidate for the multivariate model [25]. Data were completed for approximately 90% of study participants. For missing data, values were imputed. The mean of the non-missing values for numeric variables was used as the estimate of missing numeric data and the mode (most frequent) value was used as the estimate of missing categorical data [18,26]. Testing for linear trends was conducted by considering the ordinal exposure variables as continuous and then examining the significance of the coefficient with a z-test [18]. All statistical analyses were completed using SAS 9.1 [27].

#### Ethical Considerations

The protocol for the NOCS was approved in NL by the Human Investigation Committee (HIC) of the Faculty of Medicine of Memorial University. Permission for the use of NOCS data for the secondary analysis which forms the basis of the current study was provided by the NOCS co-principal investigators, and the study was subsequently approved by the HIC.

## Results

### Characteristics of cases and controls

A total of 702 CRC cases and 717 controls were included in the study. The case and control groups had a similar age-sex distribution with the mean ages at 60.3 years (SD: 9.4) in cases and 60.4 years (SD: 9.5) in controls, respectively. Men accounted for 60.7% of cases and 59.2% of controls. Caucasians accounted for over 94% of the sample and 93% were Canadian-born. Approximately 13% of the sample (102 cases and 92 controls) self-reported a previous diagnosis of other cancers, mostly non-melanoma skin cancer, but no significant differences existed between cases and controls.

Table 2 summarizes demographic characteristics of CRC cases and controls overall and alcohol drinkers, specifically. Significantly more cases than controls lived in rural areas, had an education of high school or less, had diabetes, smoked cigarettes, had a family history of CRC, had not received a diagnosis of high cholesterol, used laxatives and were obese. Significantly more controls than cases took aspirin and calcium supplements. Cases reported lower intake of fruits than controls.

**Table 2:** The demographic characteristics of CRC cases and controls and the prevalence rates of alcohol consumption by subgroup among cases and controls in the NL population-based case-control study of CRC in 1999-2003

Demographics	Case		Control		Case Drinkers^b^		Control Drinkers	
	
	N	%^a^	N	%^a^	N	% (95% CI)	N	% (95% CI)
Age group								

20-54	186	26.5	185	25.8	126	67.7 (± 6.7)	131	70.8 (± 6.5)

55-64	242	34.5	264	36.8	154	63.6 (± 6.0)	174	65.9 (± 5.7)

65-74	274	39.0	268	37.4	152	55.5 (± 5.9)	148	55.2 (± 5.9)

Sex								

Female	276	39.3	293	40.9	97	35.1 (± 5.6)	114	38.9 (± 5.6)

Male	426	60.7	424	59.1	335	78.6 (± 3.9)	339	80.0 (± 3.9)

Region		*						

Urban	302	43.0	353	49.2	196	64.9 (± 5.4)	237	67.1 (± 4.9)

Rural	400	57.0	364	50.8	236	59.0 (± 4.8)	216	59.3 (± 5.0)

Education		***						

High school or less	446	63.53	349	48.68	251	56.2 (± 4.5)	201	57.6 (± 5.2)

College+	256	36.47	368	51.32	181	70.7 (± 5.6)	252	68.5 (± 4.8)

Marital status								

Married	540	76.92	579	80.75	364	64.9 (± 4.9)	382	65.9 (± 3.8)

Single/div./sep./wid.	162	23.08	138	19.25	86	53.1 (± 7.7)	71	51.4 (± 8.3)

Family history of CRC		***						

No	540	76.92	614	85.63	342	63.3 (± 4.1)	394	64.1 (± 3.8)

Yes	162	23.08	103	14.37	90	55.5 (± 7.7)	59	57.2 (± 9.5)

Diabetes		***						

No	555	79.06	623	86.89	97	35.1 (± 5.6)	114	38.9 (± 5.6)

Yes	147	20.94	94	13.11	335	78.6 (± 3.9)	339	80.0 (± 3.9)

Any laxatives use		***						

No	573	81.62	657	91.63	97	35.1 (± 5.6)	114	38.9 (± 5.6)

Yes	129	18.38	60	8.37	335	78.6 (± 3.9)	339	80.0 (± 3.9)

Obesity		*						

No (BMI < 30)	503	71.7	556	77.5	295	58.6 (± 4.3)	359	64.6 (± 4.0)

Yes (BMI ≥ 30)	199	28.4	161	22.5	137	68.6 (± 6.2)	94	58.4 (± 7.6)

Cigarette smoking		***						

No	201	28.6	170	37.7	70	34.9 (± 6.7)	127	47.0 (± 5.9)

Yes	501	71.4	447	62.3	362	72.3 (± 4.0)	326	72.9 (± 4.1)

Cholesterol level		**						

Low	494	70.4	451	62.9	303	61.3 (± 4.3)	286	63.4 (± 4.4)

High	208	29.6	266	37.1	129	62.0 (± 6.6)	167	62.8 (± 5.8)

Aspirin		*						

No	522	74.4	492	68.6	306	58.6 (± 4.2)	306	62.2 (± 4.3)

Yes	180	25.6	225	31.4	126	70.0 (± 6.7)	147	65.3 (± 6.2)

Fruits daily		***						

≤ 2 servings	519	73.9	471	65.7	312	60.1 (± 4.2)	295	62.6 (± 4.3)

3+ servings	183	26.1	246	34.3	120	65.6 (± 6.9)	158	64.0 (± 7.8)

Calcium pills/tablets		***						

No	608	86.6	568	79.2	386	63.5 (± 4.8)	383	67.4 (± 3.8)

Yes	94	13.4	149	21.8	46	48.9 (± 10.1)	70	47.0 (± 8.0)

Total	702	100.0	717	100.0	432	61.5 (± 3.6)	453	63.2 (± 3.6)

^a^Column% and *X*^2^: *P < 0.05 **P < 0.01 ***P < 0.001

^b^Number, percentage of drinkers and 95% CI of the percentage

### Colorectal cancer risk and alcohol intake

Table 3 presents the unadjusted and adjusted ORs and their corresponding 95% CIs of colon cancer, rectal cancer and CRC with alcohol intake among men and women combined. There were no statistically significant associations between alcohol intake and risks of CRC overall or when stratified by subsite in the colon or rectum. Drinking less than 20 years tended to slightly decrease the risk of rectal cancer.

**Table 3:** The unadjusted OR and adjusted OR of CRC and the corresponding 95% CI for total alcoholic drink by cancer location among males and females in the NL population-based case-control study of CRC in 1999-2003

Alcoholic drink	Control (N = 717)	Colon Cancer			Rectal Cancer			Colorectal Cancer		
		
		N = 470	OR^a ^& 95% CI	OR^b ^& 95% CI	N = 232	OR^a ^& 95% CI	OR^b ^& 95% CI	N = 702	OR^a ^& 95% CI	OR^b ^& 95% CI
Alcohol		**								

Non-drinker^c^	264	189	1.0		81	1.0		270	1.0	1.0

Drinker^c^	453	281	0.9 0.7-1.1	1.0 0.7-1.4	151	1.1 0.8-1.5	0.9 0.6-1.3	432	0.9 0.7-1.2	1.0 0.7-1.3

Types of drinks		**								

1-2	339	213	0.9 0.7-1.1	1.0 0.7-1.3	122	1.2 0.9-1.6	0.9 0.6-1.4	335	1.0 0.8-1.2	1.0 0.7-1.3

3+	114	68	0.8 0.6-1.2	1.0 0.7-1.6	29	0.8 0.5-1.3	0.7 0.4-1.3	97	0.8 0.6-1.2	0.9 0.6-1.4

# of drink years										

1-19	111	58	0.7 0.5-1.1	0.8 0.6-1.3	20	0.6 0.3-1.0	0.5 0.3-0.9*	80	0.7 0.5-0.9*	0.7 0.5-1.0

20-39	246	154	0.9 0.7-1.2	1.0 0.7-1.5	93	1.2 0.9-1.7	1.0 0.7-1.6	247	1.0 0.8-1.3	1.0 0.8-1.4

40+	96	69	1.0 0.7-1.4	1.1 0.7-1.7	38	1.3 0.8-2.0	1.1 0.7-2.0	107	1.1 0.8-1.5	1.1 0.8-1.7

# of drinks daily		*								

1-2	287	153	0.7 0.6-1.0	0.9 0.7-1.2	88	1.0 0.7-1.4	0.9 0.6-1.3	241	0.8 0.6-1.1	0.9 0.7-1.2

3-4	68	41	0.8 0.5-1.3	0.9 0.6-1.5	19	0.9 0.5-1.6	0.6 0.3-1.2	60	0.9 0.6-1.3	0.8 0.5-1.3

5+	98	87	1.2 0.9-1.8	1.5 0.9-2.2	44	1.5 0.9-2.3	1.1 0.6-1.8	131	1.3 0.9-1.8	1.3 0.9-1.9

^a^OR unadjusted estimates for alcoholic drink

^b^OR estimates for alcoholic drink from binary models adjusted for age, sex, rural/urban, education, marriage, family history of colorectal cancer, diabetes, cholesterol, aspirin, fruits, BMI, laxatives and calcium and random effect of census area

^c^Non-drinker = non-drinkers and light drinkers who drank less than one drink per day; Drinker = drinkers who ever consumed any alcoholic beverages once a week for 6 months or longer

*X*^2 ^or Wald test or Cochran-Armitage test for trend: *P < 0.05 **P < 0.01 ***P < 0.001

### Colorectal cancer risk and alcohol intake by sex

Table 4 presents the unadjusted and adjusted ORs and 95% CIs for the associations between alcohol intake and CRC risk among men and women separately. Similar to the combined sex results, the stratified results do not show any statistically significant associations between alcohol intake and CRC risk.

**Table 4:** The unadjusted OR and adjusted OR of CRC and the corresponding 95% CI for total alcoholic drink among males and females in the NL population-based case-control study of CRC in 1999-2003

Alcoholic drink	Male					Female				
	**Case/Control**	**OR^a ^& 95% CI**		**OR^b ^& 95% CI**		**Case/Control**	**OR^a ^& 95% CI**		**OR^b ^& 95% CI**	

Total sample	426/424					276/293				

Alcohol										

Non-drinker^c^	91/85	1.0		1.0		179/179	1.0		1.0	

Drinker^c^	335/339	0.9	0.7-1.3	0.9	0.6-1.3	97/114	0.9	0.6-1.2	1.1	0.7-1.6

Types of drinks										

1-2	248244	1.0	0.7-1.3	0.8	0.6-1.3	87/95	0.9	0.6-1.3	1.2	0.8-1.9

3+	87/95	0.9	0.6-1.3	0.9	0.6-1.4	10/19	0.5	0.2-1.2	0.7	0.3-1.8

# of drinking years										

1-19	44/58	0.7	0.4-1.2	0.7	0.4-1.2	34/53	0.6	0.4-1.0	0.8	0.5-1.4

20-39	192/193	0.9	0.7-1.3	0.8	0.5-1.3	55/53	1.0	0.7-1.6	1.5	0.9-2.5

40+	99/88	0.7	0.7-1.6	1.0	0.6-1.6	8/8	1.0	0.4-2.7	1.4	0.5-4.4

# of drinks daily										

1-2	167/191	0.8	0.6-1.2	0.8	0.5-1.2	74/96	0.8	0.5-1.1	1.0	0.7-1.6

3-4	51/57	0.8	0.5-1.4	0.7	0.4-1.2	9/11	0.8	0.3-2.0	1.0	0.4-2.8

5+	117/91	1.2	0.8-1.8	1.1	0.7-1.8	14/7	2.0	0.8-5.1	1.8	0.7-5.0

^a^OR unadjusted estimates for alcoholic drink

^b^OR estimates for alcoholic drink from binary models adjusted for age, rural/urban,

education, marriage, hormone replacement (for females), family history of colorectal cancer, diabetes, cholesterol, aspirin, fruits, BMI, laxatives and calcium and random effect of census area

^c^Non-drinker = non-drinkers and light drinkers who drank less than one drink per day; Drinker = drinkers who ever consumed any alcoholic beverages once a week for 6 months or longer

### Colorectal cancer risk and alcohol intake by obesity status

Table 5 presents the unadjusted and adjusted ORs and 95% CIs for the associations between alcohol intake and CRC risk stratified by obesity status. Among non-obese participants, drinkers with low levels of alcohol intake (i.e., 1-19 years or 1-2 drinks/day) seemed to have a decreased risk of developing CRC compared to non-drinkers, with ORs of 0.6 (95% CI: 0.4-0.9) and 0.7 (95% CI: 0.5-1.0), respectively. Among obese participants, however, overall alcohol intake was positively associated with CRC risk, with OR equals to 2.2 (95% CI: 1.2-4.0). The risk of CRC appeared to linearly increase with reported increasing drinking years in obese subjects (OR:2.5, 95%CI: 1.3-5.0 for 20-39 years and OR:2.6, 95%CI:1.1-6.5 for 40+ years). Similar higher odds of CRC risk were observed among obese individuals who reported 1-2 drinks or 5+ drinks per day on average (OR:2.3,95%CI:1.2-4.3 and OR:3.7,95%CI:1.5-9.0, respectively).

**Table 5:** Association between alcohol drinking and CRC by obesity status in Newfoundland, 1999-2003

Alcoholic drink	Not Obese (BMI < 30)					Obese (BMI ≥ 30)				
	**Case/Control**	**OR^a ^& 95% CI**		**OR^b ^& 95% CI**		**Case/Control**	**OR^a ^& 95% CI**		**OR^b ^& 95% CI**	

Total sample	503/556					199/161				

Alcohol	*					*				

Non-drinker^c^	208/197	1.0		1.0		62/67	1.0		1.0	

Drinker^c^	295/359	0.8	0.6-1.0	0.8	0.6-1.1	137/94	1.6	1.0-2.4 *	2.2	1.2-4.0 *

Types of drinks	**									

1-2	233/262	0.8	0.7-1.1	0.8	0.6-1.1	102/77	1.4	0.9-2.3	2.0	1.1-3.8 *

3+	62/97	0.6	0.4-0.9 ***	0.7	0.4-1.1	35/17	2.2	1.1-4.4 **	3.4	1.4-8.1 **

# of drinking years						*				

1-19	53/82	0.6	0.4-0.9 *	0.6	0.4-0.9 **	25/29	0.9	0.5-1.8	1.6	0.7-3.7

20-39	166/197	0.8	0.6-1.1	0.8	0.6-1.2	81/49	1.8	1.1-2.9 *	2.5	1.3-5.0 *

40+	76/80	0.9	0.6-1.3	0.9	0.6-1.4	31/16	2.1	1.0-4.2 *	2.6	1.1-6.5 *

P-_trend_		ns		ns					*	

# of drinks daily	*									

1-2	167/232	0.7	0.5-0.9 *	0.7	0.5-1.0 *	74/55	1.5	0.9-2.4	2.3	1.2-4.3 *

3-4	36/47	0.7	0.5-1.2	0.7	0.4-1.2	24/21	1.2	0.6-2.4	1.3	0.5-3.2

5+	92/80	1.1	0.8-1.6	1.0	0.7-1.6	39/18	2.3	1.2-4.5 *	3.7	1.5-9.0 *

P-_trend_		ns		ns					*	

^a^OR unadjusted estimates for alcoholic drink

^b^OR estimates for alcoholic drink from binary models adjusted for cigarette smoke, age, sex, education, marriage, rural/urban, family history of colorectal cancer, diabetes, cholesterol, aspirin, fruits, laxatives and calcium and random effect of census area

^c^Non-drinker = non-drinkers and light drinkers who drank less than one drink per day; Drinker = drinkers who ever consumed any alcoholic beverages once a week for 6 months or longer

*X*^2 ^or Wald test or Cochran-Armitage test for trend: *P < 0.05 **P < 0.01 ***P < 0.001. Cochran-Armitage test for trend: ns = not significant at 5%

## Discussion

The results of this population-based case-control study in NL, Canada, demonstrated that the association between alcohol intake and risk of CRC differed by obesity status. The increased risk of alcohol drinking on CRC was observed in people with obesity, and this association remained persistent regardless of how the exposure was defined.

Previous studies have suggested that alcohol consumption may increase the risk of developing CRC [4,8,28,29]. However, our study observed a higher risk of CRC for drinking alcohol in people with obesity. While we are not able to offer a conclusive statement regarding discrepancy, there are several plausible explanations. First, obesity status is a CRC risk factor usually considered as a potential confounder in most studies investigating the alcohol -CRC association. This study examined how obesity modified the effects of alcohol on CRC rather than treated it as a confounder. Thus, our findings may help, in part, reconcile some discrepancies in the literature in terms of the risk for CRC related to alcohol drinking and provide some evidence for assessing possible interactions between alcohol and obesity in future epidemiological studies. Another possible reason for this may be that NL is a founder population geographically isolated [30]. A higher proportion of CRC incidence in NL may be mainly attributed to genetic cause that may shade the roles of other environmental factors in the CRC carcinogenesis such as alcohol consumption [19,30].

The elevated risk of CRC related to alcohol consumption only observed in obese participants in this study suggests a synergistic effect between alcohol and obesity. Although the biological mechanism for the synergy is not fully understood, a similar pattern has been detected in patients with liver, esophageal, and stomach cancer [31,32]. Possible explanations for this include that alcohol is principally metabolized to acetaldehyde, an established carcinogen causing mucosal damage and cell proliferation to humans [14,33]; while obesity is characterized by a low-grade chronic inflammatory state correlated with an increase in oxidative stress [13]. In the presence of acetaldehyde (the primary metabolite of alcohol), pro-oxidative conditions, in general, produce acetaldehyde-modified protein, which has been suggested as one of the major events initiating cellular damages and causing diseases [21]. Additionally, consistent with the elevated oxidative stress, the amount of GSTA4, an important enzyme that is responsible for the proper breakdown of acetaldehyde, has been found to decrease approximately 3-4-fold in obesity [13], resulting in an increased local toxicity of acetaldehyde. The biological mechanism, however, requires further investigation, and this observation needs to be validated in other populations.

A main strength of this study is that both cases and controls were selected from population-based samples identified through Newfoundland Cancer Registry and Telephone Roll provided by Aliant Company, respectively, resulting in a relatively large sample size (702 cases and 717 controls). All participants completed three self-administered questionnaires (i.e., a PHQ, a FHQ, and a FFQ), gathering detailed information on personal history, lifestyle and dietary habits for each participant. This allowed for the comprehensive assessment of other factors that could act as possible confounders that may distort the results [19].

This study is subject to several limitations. First, research in which people may participate has greatly proliferated in the past decades, resulting in an increased reject rate in all studies [34]. Despite our best efforts, the participation rates of both cases and controls were relatively low. Case patients were more likely to respond because of a pre-existing awareness of the disease. Given the strength of the reported associations, the magnitude of the possible bias was unable to accurately estimate. It is possible that participating controls tended to have a higher socioeconomic status than the general population, which may lead to over-estimating the risk of alcohol drinking in this study. However, an analysis of the differences in demographic characteristics (only age, sex and residence in controls) between the eligible cases and controls, between participating cases and controls, between participating and non-participating cases, and between participating and non-participating controls in this study did not show evidence that non-participation greatly affected the results of the study (data not shown). Thus, possible participation bias was unlikely to fully explained observed association in this study.

Secondly, this study may be influenced by recall bias that could lead to exposure misclassification. Because the questionnaire did not distinguish respondents who never drank alcohol from those who drank less than one drink per day, the referent category (i.e., "non-drinkers") included occasional drinkers with non-drinkers. Lifetime measures might be inaccurate due to the concern about the adequacy of long-term recall after 18 years [35]. When questioned on exposure status, participants were more likely to underestimate the amount of alcohol they drank [36], which would generally bias the association towards to the null [37-39]. The validity of self-reported alcohol consumption may differ between cases and controls since the cases and controls by definition are people who differ with respect to their disease experience, and this difference may affect recall [40]. The bias caused by differential misclassification is variable [41]. However, the main protection against bias in this study was the standardization of methods. The questions were identical and presented in an identical fashion to both cases and controls. Furthermore, analyses conducted to assess the validity of self-reporting lifetime alcohol consumption in this study (data not shown) did not provide any evidence that inaccurate or differential reporting of alcohol consumption for cases and controls may have biased the association.

## Conclusion

This study conducted in the province of NL, Canada found that alcohol consumption was associated with higher risk of CRC in the presence of obesity. These observations require being validated in large prospective studies. If confirmed, the data may have implications for exploring the association between alcohol and obesity and possible mechanisms for their combined effects, and for tailoring alcohol policies and prevention strategies for obese people.

## Competing interests

The authors declare that they have no competing interests.

## Authors' contributions

JZ led the data analysis and finding interpretation, and was the writer of the first version of the manuscript. YZ contributed to the consequent writing and revising of the manuscript. PPW conceptualized, devised and actively commented on the overall research design, the results interpretation, and the paper. ZS and JS collated the manuscript for intellectual content. RW, SB, BR, JRM, PTC, PSP, the co-project investigators, sought funding for the original project, critically commented, and oversaw the scientific implementation of the study. All authors read and approved the final version.

## Pre-publication history

The pre-publication history for this paper can be accessed here:

http://www.biomedcentral.com/1471-2458/12/94/prepub
